# Molecular Identification of Sibling Species of *Sclerodermus* (Hymenoptera: Bethylidae) That Parasitize Buprestid and Cerambycid Beetles by Using Partial Sequences of Mitochondrial DNA Cytochrome Oxidase Subunit 1 and 28S Ribosomal RNA Gene

**DOI:** 10.1371/journal.pone.0119573

**Published:** 2015-03-17

**Authors:** Yuan Jiang, Zhongqi Yang, Xiaoyi Wang, Yuxia Hou

**Affiliations:** 1 Key Laboratory of Forest Protection, China State Forestry Administration, Research Institute of Forest Ecology, Environment and Protection, Chinese Academy of Forestry, Beijing, 100091, China; 2 College of Science, China Agricultural University, Beijing, China; Oklahoma State University, UNITED STATES

## Abstract

The species belonging to *Sclerodermus* (Hymenoptera: Bethylidae) are currently the most important insect natural enemies of wood borer pests, mainly buprestid and cerambycid beetles, in China. However, some sibling species of this genus are very difficult to distinguish because of their similar morphological features. To address this issue, we conducted phylogenetic and genetic analyses of cytochrome oxidase subunit I (*COI*) and 28S RNA gene sequences from eight species of *Sclerodermus* reared from different wood borer pests. The eight sibling species were as follows: *S*. *guani* Xiao *et* Wu, *S*. *sichuanensis* Xiao, *S*. *pupariae* Yang *et* Yao, and *Sclerodermus* spp. (Nos. 1–5). A 594-bp fragment of *COI* and 750-bp fragment of 28S were subsequently sequenced. For *COI*, the G-C content was found to be low in all the species, averaging to about 30.0%. Sequence divergences (Kimura-2-parameter distances) between congeneric species averaged to 4.5%, and intraspecific divergences averaged to about 0.09%. Further, the maximum sequence divergences between congeneric species and *Sclerodermus* sp. (No. 5) averaged to about 16.5%. All 136 samples analyzed were included in six reciprocally monophyletic clades in the *COI* neighbor-joining (NJ) tree. The NJ tree inferred from the 28S rRNA sequence yielded almost identical results, but the samples from *S*. *guani*, *S*. *sichuanensis*, *S*. *pupariae*, and *Sclerodermus* spp. (Nos. 1–4) clustered together and only *Sclerodermus* sp. (No. 5) clustered separately. Our findings indicate that the standard barcode region of *COI* can be efficiently used to distinguish morphologically similar *Sclerodermus* species. Further, we speculate that *Sclerodermus* sp. (No. 5) might be a new species of *Sclerodermus*.

## Introduction

*Monochamus alternatus* Hope (Coleoptera: Cerambycidae), a longhorned pine sawyer beetle, is not only considered the major pest of pine trees but also an important vector of the pinewood nematode, the causal agent of pine wilt disease [1–3]. Since its discovery in 1982 at Nanjing City, Jiangsu Province, China, the pinewood nematode has spread to 179 counties in 15 provinces (and municipalities) of China (China State Forestry Administration 2013) [4]. In the last 20 years, pine wilt disease was reported in 10 provinces of China [5]. Between 1996 and 2001, the stand volume loss caused by the pine wilt disease was more than 1 million m^3^, and the direct economic loss was estimated to be approximately above 400 billion Yuan RMB [6]. Therefore, a biological control program was initiated in 2000 to control the longhorned beetle [4]. Further, that program surveyed the natural enemies of *M*. *alternatus* [4].

Thus far, several *Sclerodermus* species are used as insect natural enemies to control the longhorned beetle in China, such as *S*. *guani*, *S*. *sichuanensis*, and *S*. *pupariae*. *S*. *guani* was used to attack *Semanotus bifasciatus* Motschulsky (Coleoptera: Cerambycidae) in Miyun, Beijing, China. The average outdoor parasitism of this pest was reduced up to 69.68% after 5 years of releasing *S*. *guani*. Further, the percentage of injury to *Platycladus orientalis* Franco declined from 22.78% to 0.49% [7]. The parasitic ability of *S*. *guani* against *Anoplophora glabripennis* Motschulsky (Coleoptera: Cerambycidae) was tested, and the results showed that the average death rates of the first, second, and third instar larvae were 100%, 92.1%, and 87.29%, respectively. The average parasitism of *S*. *guani* toward *A*. *glabripennis* in the early-stage larvae was 32.51% in a field experiment. The highest parasitism of 32.51% was noted when the ratio of pest and Bethylidae species was 1: 8–10 [8]. These findings suggest that *S*. *guani* is an important parasitic enemy that can be effectively used to prevent *M*. *alternatus* larvae; the parasitism was more than 20% in Fujian, China [9]. The parasitic ability of *S*. *sichuanensis* for attacking *M*. *alternatus* larvae on *Pinus massoniana* was significantly higher [10].

*S*. *pupariae* was described as a new parasitoid species in China by Yang Zhongqi [11]; it has a high potential to be an important natural enemy that can be used for the biological control of stem borer pests. In China, *S*. *pupariae* were reared from *Agrilus planipennis* Fairmaire (Coleoptera: Buprestidae) that attacked velvet ash (*Fraxinus velutina* Torr.), a tree native to North America. The parasitism rate in the field was 13%. Adult wasps reared from single host pupa or mature larva ranged from 24 to 56 individuals. Laboratory rearing resulted in an emerging adult female to male ratio of 22:1 [11]. Results of the biological characterization showed that many kinds of forest borer pest larvae, such as those of Buprestidae *Agrilus auriventris* Saunders, *A*. *mali* Matsumura, and *A*. *zanthoxylumi* Hou, and those of Cerambycidae *Stenhomalus complicates* Gressitt, *A*. *glabripennis*, *M*. *alternatus*, and *Thyestilla gebleri* Faldermann, could be used as hosts by *S*. *pupariae* during their feeding and progeny development phases. *S*. *pupariae* has a long life and high fecundity and diffusion capacity; therefore, it is a good insect natural enemy for the biocontrol of borer pests [12]. In 2010, Wang indicated that *S*. *pupariae* was parasitic to *Massicus raddei* Blessig (Coleoptera: Cerambycidae) larvae and found that its feeding on the hosts killed great number of pests; therefore, it has a remarkable prospect in biological control [13]. Because *S*. *pupariae* has high host-searching and host-attacking abilities and produces high proportion of females, as well as alate females (advantageous for dispersing after releasing in biocontrol programs), it has a high potential to be used as a biocontrol agent against the emerald ash borer [11].

The progress of a biological control program for longhorned beetles has been monitored for more than ten years by our team; we identified many natural insect enemies that could be used to control the longhorned beetle. Several species of *Sclerodermus* are currently the most important natural insect enemies against forest wood borer pests, mainly buprestid and cerambycid beetles, in China. Several putative species of *Sclerodermus* were identified and reared from different stem borer pests at our Key Laboratory of Forest Protection, such as *Sclerodermus* sp. (No. 1) parasitic on the pupae and larvae of *Agrilus mali*, *Sclerodermus* sp. (No. 2) parasitic on the larvae of *Chalcophora japonica* Gory, *Sclerodermus* sp. (No. 3) parasitic on the larvae of *Chrysobothris* sp. (Coleoptera: Buprestidae), *Sclerodermus* sp. (No. 4) parasitic on the larvae of *Sphenoptera* sp., and *Sclerodermus* sp. (No. 5) parasitic on the larvae and pupae of *M*. *alternatus*. These species were artificially reared in large numbers in the laboratory. All these *Sclerodermus* putative species were collected from different geographical or climatic environments. Furthermore, they were collected from different host plant species infected by different host pests. Therefore, we speculate that there might be some new species among these *Sclerodermus* putative species collected from the field. However, distinguishing these *Sclerodermus* putative species is difficult for many applied entomologists because most scale insects are small, and closely related species might be very similar morphologically.

At present, DNA barcoding is a new approach for species-level identification. Although considerable biological research depends on species diagnoses, taxonomic expertise is lacking. Sustainable identification ability involves the construction of systems that employ DNA sequences as taxon barcodes. The mitochondrial gene cytochrome c oxidase I (*COI*) could serve as the core of a global bioidentification system for animals. In some cases, this has shown to have a 100% success rate in correctly identifying specimens [14]. Thus, the *COI* identification system is a reliable, cost-effective, and accessible solution to the current problem of species identification.

The family Bethylidae belongs to the aculeate (stinging) Hymenoptera that includes familiar bees, social wasps, and ants [15]. It is placed in the superfamily Chrysidoidea, members of which are almost exclusively parasitoids [16]. Within the superfamily, the Bethylidae are possibly the most species-rich family, with over 2,000 species that have been described [17]. They are globally distributed and develop as ectoparasitoid on larvae, and occasionally, pupae, of Coleoptera and Lepidoptera species, which they permanently paralyze with a sting (idiobiosis) [18]; a few species are found on other hosts, including Hymenoptera species [19]. Gordh and Móczár [17] published a catalog of the world species of Bethylidae, including *Sclerodermus*. Evans [20, 21] studied the North American Bethylidae, including *Sclerodermus*. Lanes and Azevedo [22] listed 71 species of *Sclerodermus* worldwide when they studied the phylogeny and taxonomy of Sclerodermini. Although the phylogeny of Bethylidae was constructed in 2010 [23], there were only two samples of Sclerodermini; in this study, we further identified several *Sclerodermus* species and putative species *Sclerodermus* nos. 1–5 as well as other species of Bethylidae, sequences of which were recently obtained from GenBank. The phylogeny of Bethylidae and taxonomic classification of *Sclerodermus* were investigated in this study. However, most importantly, we aimed to use molecular methods to identify *Sclerodermus* putative species that are difficult to distinguish morphologically. Further, we provided reliable evidence for traditional morphological identification.

## Material and Methods

### Ethics statement

Our study did not involve the use of any non-human primates. Further, we confirm that our field studies did not involve endangered or protected species (i.e., the specimens sampled were *Sclerodermus* wasps (Hymenoptera: Bethylidae)). The putative species of *Sclerodermus* spp. (Nos.1–5) were collected from Tongchuan, Ningbo, Kuandian, Bayanhaote, and Kunming, respectively, China. All the fields from where the samples were collected were managed by the local Forest Pest and Disease Control Stations, and the secretaries of the respective stations provided permission to conduct field studies. Finally, no permits were required for the described study, which complied with all the relevant regulations.

### Specimen sampling

In total, 136 individuals were collected from different progeny, and 15 individuals of each putative species were reared at the Chinese Academy of Forestry, Beijing, China. Further, *S*. *guani*, *S*. *sichuanensis*, and *S*. *pupariae* were collected from Beijing Xishan Forest Farm. Some *S*. *sichuanensis* samples were obtained from Chongqing Academy of Forestry. Table 1 provides an overview of the origin hosts, origin place from where the specimens were collected, and rearing conditions for each putative species, as well as the number of species that have been named and published or are unnamed. In this study, all putative species were reared artificially in the laboratory and were descendants of origin species. For each species, the genomic DNA was extracted from an adult female by using a DNA extraction kit (Tiangen Biotech, Beijing, China). The specimens for phylogenetic analysis of 28S ribosomal RNA gene and thirteen species of other three closely related families were obtained as an outgroup from GenBank; these are listed in Table 2.

**Table 1:** Description of samples used in this study.

Insect species	Origin host	Origin place from where the specimens were collected (coordinates for the test sites)	Rearing laboratories used for the samples	No. of Species	Species names if published
*S*. *guani*	Larvae of *Semanotus sinoauster* and *Saperda populnea*	Shantou (116°14′-117°19′E, 23°02′-23°38′N) and Guangzhou (113°17'E, 23°8'N) of Guangdong Province, Xuzhou (116°22′-118°40′E, 33°43′-34°58′N) of Jiangsu Province, Yulin (107°28′-111°15′E, 36°57′-39°34′N) of Shaanxi Province, Taiyuan (111°30′-113°09′E, 37°27′-38°25′N) of Shanxi Province, Wangdu (115°01′16″-115°18′13″E, 38°30′46″-38°48′30″N) of Hebei Province, Shandong Province, Henan Province, China	CAF, Beijing, China	15 (Sg 1 to 15)	Named and published in 1983
*S*. *guani*			Xishan Forest Farm, Beijing, China	6 (Sg 16 to 21)	Named
*S*. *sichuanensis*	Larvae and pupae of *Semanotus sinoauster*	Luxian (105°10′50″-105°45′30″E, 28°54′40″-29°20′00″N) of Sichuan Province, China	CAF, Beijing, China	15 (Ss 1 to 15)	Named and published in 1995
*S*. *sichuanensis*			Xishan Forest Farm, Beijing, China	3 (Ss 16 to 18)	Named
*S*. *sichuanensis*			Chongqing Academy of Forestry, China	6 (Ss 19 to 24)	Named
*S*. *pupariae*	*Agrilus planipennis*	Guangang Forest Farm of Tianjin (117°29′E, 38°56°N) Municipality, China	CAF, Beijing, China	15 (Sp 1 to 15)	Named and published in 2012
*S*. *pupariae*			Xishan Forest Farm, Beijing, China	1 (Sp16)	Named
*Sclerodermus* sp. (No. 5)	*Monochamus alternatus*	Kunming (102°42′21″E, 25°03′25″N), Yunnan Province, China	CAF, Beijing, China	15 (No. 5 1 to 15)	Unnamed
*Sclerodermus* sp. (No. 2)	*Chalcophora japonica*	Ningbo (121°04′E, 29°57′N) of Zhejiang Province, China	CAF, Beijing, China	15 (No. 2 1 to 15)	Unnamed
*Sclerodermus* sp. (No. 1)	*Agrilus mali*	Tongchuan (108°58′37″E, 34°55′18″N) of Shaanxi Province, China	CAF, Beijing, China	15 (No. 1 1 to 15)	Unnamed
*Sclerodermus sp*. (No. 3)	*Chrysobothris* sp.	Kuandian (125°11′E, 44°45′N) of Liaoning Province, China	CAF, Beijing, China	15 (No. 3 1 to 15)	Unnamed
*Sclerodermus* sp. (No. 4)	*Sphenoptera* sp.	Bayanhaote (105°37′19″E, 38°51′51″N) of Inner Mongolia, China	CAF, Beijing, China	15 (No. 4 1 to 15)	Unnamed

**Table 2:** The specimens used for phylogenetic analysis of 28S ribosomal RNA gene.

Family	Subfamily (Tribe)	Species	Genbank
Bethylidae	Scleroderminae	*Sclerodermus guani*	This study
		*Sclerodermus sichuanensis*	This study
		*Sclerodermus pupariae*	This study
		*Sclerodermus* sp. (No. 1)	This study
		*Sclerodermus* sp. (No. 2)	This study
		*Sclerodermus* sp. (No. 3)	This study
		*Sclerodermus* sp. (No. 4)	This study
		*Sclerodermus* sp. (No. 5)	This study
		*Sclerodermus* sp.	GU213956
		*Allobethylus* sp.	GU213960
		*Prorops nasuta*	GU213957
		*Prorops* sp.	GU213959
		*Cephalonomia hyalinipennis*	GU213961
		*Cephalonomia stephanoderis*	GU213962
		*Cephalonomia formiciformis*	KC762949
		*Cephalonomia hypobori*	KC762951
		*Cephalonomia gallicola*	KC762950
		*Plastanoxus* sp.	GU213937
	Mestiinae	*Sulcomesitius* sp.	GU213958
	Epyrinae	*Anisepyris* sp.	GU213964
		*Anisepyris subviolaceus*	GU213965
		*Holepyris* sp.	GU213970
		*Laelius pedatus*	GU213963
		*Epyris* sp. 1	GU213969
		*Epyris* sp. 2	GU213967
		*Epyris* sp. 3	GU213968
		*Epyris* sp. 4	GU213966
	Pristocerinae	*Foenobethylus emiliacasellae*	GU213952
		*Pseudisobrachium* sp.	GU213955
		*Pristocera* sp. 1 EMP-2008	EU367152
		*Pristocera* sp. 1 MC-2010	GU213954
		*Trichiscus* sp.	GU213953
		*Dissomphalus* sp. 1	GU213949
		*Dissomphalus* sp. 2	GU213951
		*Dissomphalus* sp. 3	GU213950
	Bethylinae	*Bethylus cephalotes*	GU213939
		*Bethylus fuscicornis*	GU213940
		*Goniozus* (sensu strictu) sp. 1	GU213942
		*Goniozus* (sensu strictu) sp. 2	GU213943
		*Goniozus* (Parasierola) sp.	GU213941
		*Goniozus legneri*	GU213948
		*Goniozus nephantidis*	GU213947
		*Odontepyris* sp. 1	GU213944
		*Odontepyris* sp. 2	GU213945
		*Odontepyris* sp. 3	GU213946
Braconidae	Alysiinae	*Aphaereta genevensis*	GU213932
	Blacinae	*Blacus* sp.	GU213930
	Microgastrinae	*Cotesia* sp.	GU213931
Chrysididae	Amiseginae	*Bupon* sp.	GU213974
	Chrysidinae (Chrysidini)	*Chrysis* sp.	GU213972
		*Chrysis ruddii*	GU213973
	Chrysidinae (Elampini)	*Omalus* sp.	GU213935
	Chrysidinae	*Holophris* sp.	GU213936
	Cleptinae	*Cleptes semiauratus*	GU213975
	Loboscelidiinae	*Loboscelidia* sp.	GU213971
Dryinidae		*Anteon infectum*	GU213934
Embolemidae		*Embolemus* sp.	GU213933
Ichneumonidae	Rhyssinae	*Rhyssa persuasoria*	GU213938

### DNA extraction, amplification, and sequencing

Total DNA was extracted from fresh individuals by using TIANamp Genomic DNA Kit (Tiangen), following manufacturer’s protocols. Universal primers C1-J-1718 (5′-GGAGGATTTGGAAATTGATTAGTTCC-3′) and C1-N-2329 (5′-ACTGTAAATATATGATGATGAGCTCA-3′) [24] were first used to amplify 600-bp of mitochondrial *COI* of eight *Sclerodermus* putative species. PCR conditions were as follows: initial step of 5 min at 95°C, followed by 35 cycles of 45 s at 95°C, 45 s at 50°C, and 30 s at 72°C, and a final extension of 3 min at 72°C; the reactions were conducted using an Eppendorf Mastercycler Pro S (Germany). All 136 samples could be successfully amplified using the universal primers. The 28S ribosomal gene was amplified using primers 28SD2F1 (5′-CGTGTTGCTTGATAGTGCAGC-3′) [25] and 28SD3R (5′-TAGTTCACCATCTTTCGGGTC-3′) [26]. All PCRs were processed in a 25 μL volume containing 1.5 μL DNA template, 3 μL 10× *Taq* buffer, 2 μL dNTP mixture (2.5 mM), 1 μL primer (10 pmol/μL), and 0.5 μL *Taq* DNA polymerase (2.5 U/μL) (Tiangen, ET101). PCRs were conducted using TECHNE Techgene thermocycler by using the following profile: an initial denaturation for 2 min at 94°C, followed by 29 cycles of denaturation at 94°C (30 s), annealing at 50°C (45 s), and extension at 72°C (90 s), and final extension for 10 min at 72°C. Products were visualized on 1% agarose gel; photos were obtained using MutiImage Light Cabinet and saved. The PCR products were directly sequenced by SANBO Company. Sequences were aligned using DNAMAN. Sequence divergences were calculated using the K2P distance model. Neighbor-joining (NJ) trees of *COI* and D2 expansion segments of 28S were reconstructed using MEGA5.1. *Cephalonomia gallicola* Ashmead, 1887 (Bethylidae: Scleroderminae: *Cephalonomia*) [27, 28] was chosen as the outgroup for constructing the NJ trees.

All sequences were entered into the GenBank database (*COI* DNA sequences: GenBank Accessions KM649832-KM649967. 28S DNA sequences: GenBank Accessions KM649696-KM649831).

## Results

### DNA sequence analyses

In this study, 136 individuals from eight putative species of the genus *Sclerodermus* were used. The samples per putative species ranged from one to twenty-four, with an average (mode) of fifteen. The sequence of a 594-bp *COI* fragment was obtained from all *Sclerodermus* individuals after deleting the terminal ambiguous part of the aligned data. There was 100% sequence identity between *S*. *guani* and *S*. *sichuanensis*. For *COI* data, low G-C content was found in all species, averaging to about 30.0%. Sequence divergences (K2P) between congeneric species averaged 4.5%, whereas intraspecific divergences averaged 0.09%. Furthermore, no insertions or deletions were found in DNA sequences, and no stop codons were found when amino acids were translated, implying that all amplified sequences coded for functional *COI*. The 594-bp nucleotide fragment of *COI* corresponded to 198 codons. Interestingly, there was 3.0% interspecific divergence between the *COI* regions of *S*. *guani* and *S*. *pupariae*, whereas the translated amino acid sequences of the two species were not different. Further, the 1.4% divergence (K2P) between *S*. *guani* and *Sclerodermus* sp. (No. 3) did not lead to a difference in amino acid sequences. Generally, the maximum interspecific divergence between *S*. *guani* and *Sclerodermus* sp. (No. 5) was 16.5%; this translated into a difference of two amino acids.

Comparison of the sequences of five unknown putative species with those of three known species (*S*. *guani*, *S*. *sichuanensis*, and *S*. *pupariae*) revealed that the sequence similarity of *Sclerodermus* sp. (No. 4) and *S*. *pupariae* was 100%. However, the lowest sequence similarity of about 85% was found between *Sclerodermus* sp. (No. 5) and the three known species (Table 3).

**Table 3:** *COI* gene sequence data analysis of the five unnamed *Sclerodermus* sp. together with that of the three known species.

	Conserved sites			Variable sites			Genetic distance			Sequence similarity		
	*Sg*	*Ss*	*Sp*	*Sg*	*Ss*	*Sp*	*Sg*	*Ss*	*Sp*	*Sg*	*Ss*	*Sp*
(No. 1)	590	590	576	4	4	18	0.007	0.007	0.030	99.33%	99.33%	97.14%
(No. 2)	591	591	573	3	3	19	0.006	0.006	0.032	99.49%	99.49%	96.79%
(No. 3)	586	586	574	8	8	20	0.014	0.014	0.034	98.65%	98.65%	96.80%
(No. 4)	577	577	594	17	17	0	0.030	0.030	0.000	97.14%	97.14%	100%
(No. 5)	507	507	508	87	87	86	0.165	0.165	0.161	85.35%	85.35%	85.69%

*Sg*: *Sclerodermus guani*; *Ss*: *Sclerodermus sichuanensis*; *Sp*: *Sclerodermus pupariae*; Nos. 1 to 5: *Sclerodermus* spp. (Nos. 1–5)

**Fig 1:**
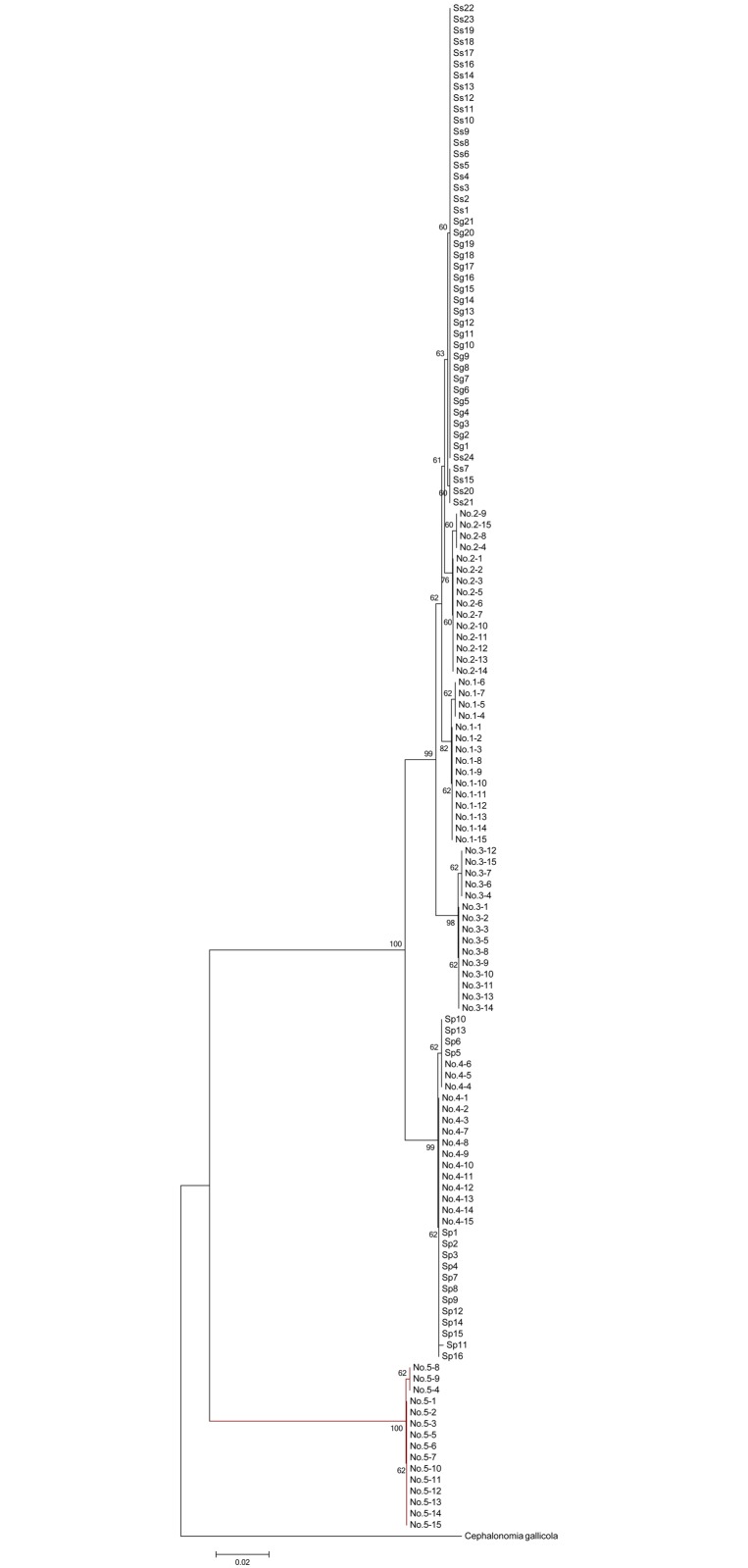
Neighbor-joining tree of the *COI* sequences of the sibling species of the genus *Sclerodermus* developed using Kimura-2-parameter distance. Bootstrap values for each haplogroup were calculated using MEGA5.1 with 1,000 replicates. *Cephalonomia gallicola* (Bethylidae; Epyrinae; Cephalonomia) was chosen as the outgroup.

In the *COI* region, the averaged frequency of adenine (A) and thymine (T) was high (A = 30.9%, C = 18.4%, G = 11.6%, T = 39.1%). The G-C frequency was lower at the third codon position (mean, 14.8% for all individuals) than that at the first and second positions (mean, 36.4% and 38.9%, respectively). The nucleotide frequency of the three codon positions in the eight species is shown in Table 4. The G-C frequency at the third codon position (mean, 18.2% for all individuals) was the highest for *Sclerodermus* sp. (No. 5), whereas that of *S*. *pupariae* and *Sclerodermus* spp. (Nos. 3 and 4) was 15.1%, which was higher than that of the others (14.1%). Further, the G-C frequency at the first codon position of *Sclerodermus* sp. (No. 3) was slightly higher than that of the others. The 594-bp region of *COI* mtDNA was obtained for the 136 individuals; it included 491 conserved sites, 103 variable sites, 102 parsimony-informative sites, one singleton site, 375 zero-fold degenerate sites, 115 two-fold degenerate sites, and 81 four-fold degenerate sites. The *COI* sequences showed interspecific divergence of 0–17.4%, with a mean divergence of 4.5%. Intraspecific *COI* sequence divergences were 0–0.1%, with a mean divergence of 0.075% (Fig. 1). The maximum K2P distance was found between *Sclerodermus* sp. (No. 5) and *Sclerodermus* sp. (No. 3; 17.4%). Unexpectedly, the minimum distance was between *S*. *guani* and *S*. *sichuanensis* (0%) and, as expected, *Sclerodermus* sp. (No. 4) and *S*. *pupariae* clusters showed the minimal level of divergence since they might be one species.

**Table 4:** Nucleotide frequency of the three codon positions in the eight species.

	T-1	C-1	A-1	G-1	T-2	C-2	A-2	G-2	T-3	C-3	A-3	G-3
*Sg*	30.8	17.2	32.8	19.2	43.4	24.2	17.7	14.6	43.9	13.6	41.9	0.5
*Ss*	30.8	17.2	32.8	19.2	43.4	24.2	17.7	14.6	43.9	13.6	41.9	0.5
*Sp*	30.8	17.2	32.8	19.2	43.4	24.2	17.7	14.6	42.4	14.6	42.4	0.5
(No. 1)	30.8	17.2	32.8	19.2	43.4	24.2	17.7	14.6	43.9	13.6	41.9	0.5
(No. 2)	30.8	17.2	32.8	19.2	43.4	24.2	17.7	14.6	44.9	12.6	41.4	1.0
(No. 3)	30.3	17.7	32.8	19.2	43.4	24.2	17.7	14.6	43.9	12.6	41.4	2.0
(No. 4)	30.8	17.2	32.8	19.2	43.4	24.2	17.7	14.6	42.4	14.6	42.4	0.5
(No. 5)	31.3	16.7	32.8	19.2	43.9	24.2	17.2	14.6	38.4	16.2	43.4	2.0

Abbreviations are the same as those in Table 3

NJ analysis of the *COI* sequences revealed that the 136 samples were split into six haplogroups with high bootstrap values (Fig. 1). *S*. *guani* and *S*. *sichuanensis* were merged into one haplogroup, whereas *S*. *pupariae* and *Sclerodermus* sp. (No. 4) were merged into another haplogroup. *S*. *guani* and *S*. *sichuanensis*, *Sclerodermus* sp. (No. 2), and *Sclerodermus* sp. (No. 1) were separated into three clusters, indicating that they were closely related. The remaining putative species, *S*. *pupariae* and *Sclerodermus* sp. (No. 4), *Sclerodermus* sp. (No. 5), and *Sclerodermus* sp. (No. 3) formed three distinct clusters, with interspecific divergence ranging from 3.4% to 17.4%.

**Fig 2:**
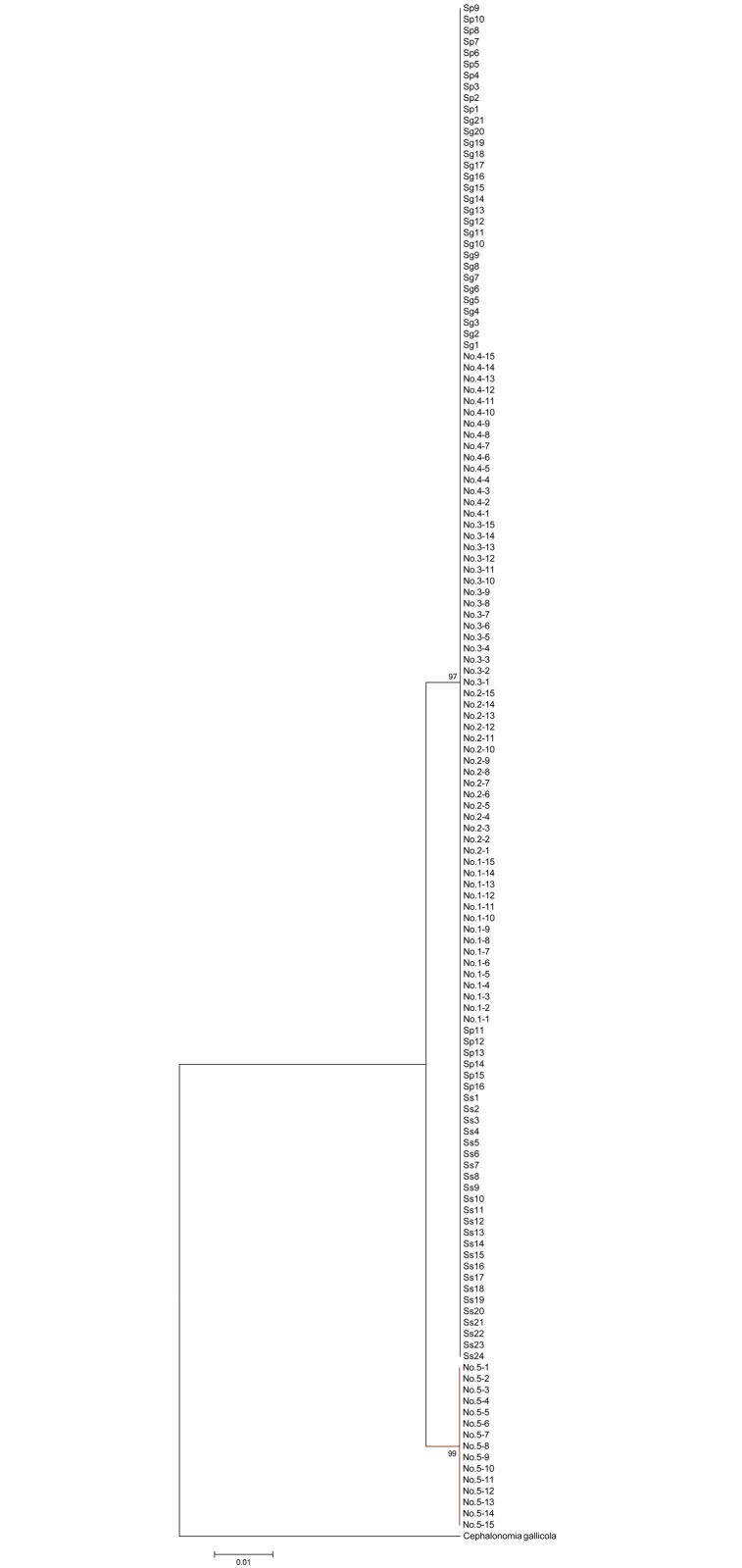
Neighbor-joining tree for the 28S of the sibling species of the genus *Sclerodermus* developed using Kimura-2-parameter distance. Bootstrap values for each haplogroup were calculated using MEGA5.1 with 1,000 replicates. *Cephalonomia gallicola* (Bethylidae; Epyrinae; Cephalonomia) was chosen as the outgroup.

The 536-bp sequence of the final alignment of the 28SrDNA gene was obtained for the 136 individuals; this sequence included 523 conserved sites, six variable sites, and six parsimony-informative sites. The NJ tree of the 28S sequence (Fig. 2) almost merged into one cluster, except that the samples of *Sclerodermus* sp. (No. 5) were clustered together. Further, the 28S gene sequence similarity of *S*. *guani*, *S*. *sichuanensis*, *S*. *pupariae*, and *Sclerodermus* spp. (Nos. 1–4) was 100%, except for *Sclerodermus* sp. (No. 5) which had 99.18% sequence similarity with those of others. Further, the K2P distance between *Sclerodermus* sp. (No. 5) and the other seven species was 1.1%.

### 28S phylogeny of the Bethylidae species

**Fig 3:**
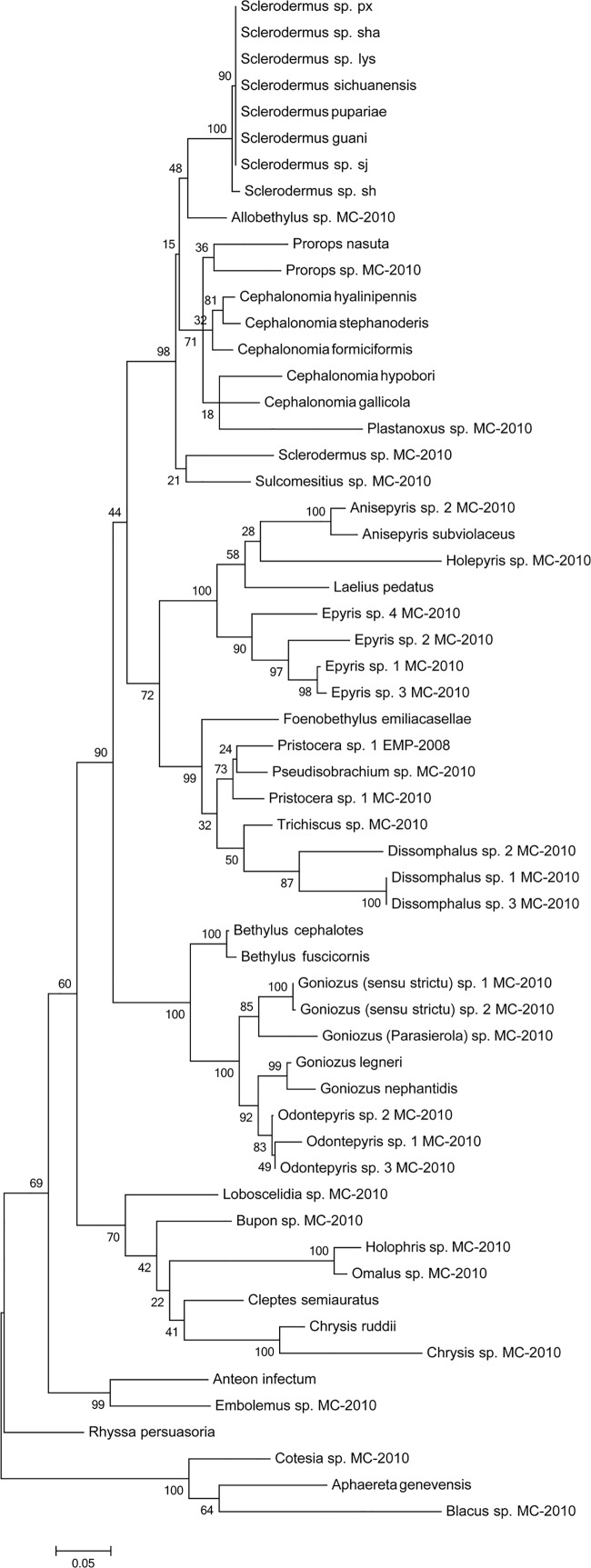
The 28S phylogeny of Bethylidae. The tree was derived using the maximum likelihood method.

Phylogenetic analysis was performed on a partial 28S ribosomal RNA D2 gene. The maximum likelihood (ML) tree indicated that Bethylidae are strongly monophyletic (90% ML bootstrap percentage (mlBP)). Of the subfamilies, Bethylinae are strongly monophyletic (100% mlBP) and sister to the other commonly recognized subfamilies (Fig. 3). Of the other subfamilies, Pristocerinae were found to be monophyletic (99% mlBP), with Foenobethylus clustering within the group [27, 28], and Pristocerinae was sister to Epyrini [23]. Only Mesitiinae species were strongly included as clusters in a group with Sclerodermini. Sclerodermini and Cephalonomini [23] were strongly supported as monophyletic (98% mlBP). The relationships between the three clades Epyrini, Pristocerinae, and (Sclerodermini + Cephalonomini + Mesitiinae) were equivocal. Species in the same genus clustered together, although they were not always strongly supported to be monophyletic (e.g., *Sclerodermus* sp. MC-2010 and *Sclerodermus* in this study; Fig. 3).

## Discussion

With the development of modern molecular biological techniques, specific DNA fragments can be used for species identification for determining the general phylogenetic relations and taxonomic status. Gene fragments or site-specific information is the auxiliary tool to distinguish species and has been used in the identification of animal and plant species.

In the present study, *COI* barcode sequences were used for the identification of several *Sclerodermus* species for the first time, to our knowledge. Each of the eight *Sclerodermus* samples had a distinct *COI* sequence, and hence, they could be distinguished from conspecifics by using the DNA barcode method. The average intraspecies K2P distance was estimated to be only 0.09%, whereas it was about 4.5% among congeneric species. This distance is considerably lesser than that for the six *Ceroplastes* species (Hemiptera: Coccidae) from China [29]. This could be attributed to the fact that our intraspecies samples were reared in the laboratory and not collected from different locations. However, the sequence divergences between *Sclerodermus* sp. (No. 5) and any congeneric species was more than 16.0%, whereas the average intraspecies K2P distance for *Sclerodermus* sp. (No. 5) was only 0.1%. The *COI* gene marker could be used for phylogenetic analysis and for the identification of cryptic species. Different vertebrate species commonly show a value close to 3% K2P distance for *COI*, a threshold adopted for Lepidoptera [14]. Thus, more than 16% sequence divergences for *Sclerodermus* sp. (No. 5) might be strong evidence for a new *Sclerodermus* species. Similarly, the 594-bp nucleotide fragment of *COI* corresponding to 198 codons led to a difference of two different amino acids between *Sclerodermus* sp. (No. 5) and the others. Further, for the 28S gene, although all the other seven species were completely identical with each other, the sequence similarity between *Sclerodermus* sp. (No. 5) and others was 99.18%. The NJ tree of *COI* also indicated that the 15 individuals of *Sclerodermus* sp. (No. 5) were strongly monophyletic (100% (mlBP)). The 28S NJ tree also yielded the same result (99% mlBP). Both the molecular phylogenetic trees suggested that *Sclerodermus* sp. (No. 5) differentiated the earliest among the species belonging to Sclerodermini. This is also supported by the fact that there was 99.18% sequence similarity between *Sclerodermus* sp. (No. 5) and the remaining species. However, determining whether *Sclerodermus* sp. (No. 5) is a new species on the basis of the morphological characteristics identified by Prof. Zhongqi Yang [11] is important in order to develop a biological control method for the newly discovered parasitic natural enemy. Studies on the biological characteristics and behavior of the new species might help in determining whether it is an important parasitoid and a biological agent for the control of larvae and pupae of *M*. *alternatus*.

The *COI* gene sequences for *S*. *guani* and *S*. *sichuanensis* were exactly the same. Thus, we speculated that the samples for the individuals of the two species had been mixed with each other. Subsequently, we found a laboratory where *S*. *guani* and *S*. *sichuanensis* species were reared over a long time; *S*. *guani* and *S*. *sichuanensis* samples were then obtained from the Xishan Forest Farm in Beijing and Chongqing Academy of Forestry Labs, respectively. However, the results of gene sequence analysis were still the same. Further studies might be necessary to explain this phenomenon.

Moreover, there was 100% sequence similarity between *S*. *pupariae* and *Sclerodermus* sp. (No. 4). That might suggest that *Sclerodermus* sp. (No. 4) was indeed *S*. *pupariae*. In 2009, Xiaoyi Wang had used *S*. *pupariae* to control *Sphenoptera* sp. in Bayanhaote, Inner Mongolia. We also collected *Sphenoptera* sp. from the same location in 2012 and reared *Sclerodermus* sp. (No. 4) from *Sphenoptera* sp. in our laboratory. Therefore, we could speculate that they are the same species. However, whether they should be considered as the same species needs further research. Furthermore, *Sclerodermus* sp. (No. 4) was found in Alashan, Inner Mongolia, suggesting that they could successfully survive and reproduce there. Thus, artificially enlarging their settlement range might contribute substantially to their biological control.

Our results that Bethylidae and Bethylinae as well as Pristocerinae are strongly monophyletic, and that Pristocerinae are clustered with *Foenobethylus* and Mesitiinae are clustered with Scleroderminae, are consistent with the findings of Martin Carr in 2010 [23]. Further, the relationships between the three clades Epyrini, Pristocerinae, and Mesitiinae + Scleroderminae are close, supporting the fact that they might be monophyletic. However, in our study, *Sclerodermus* was found to be more closely related to *Cephalonomia* than to *Sclerodermus* sp. MC-2010, which thus, was not considered as the sister of *Sclerodermus*. This discrepancy could be attributed to the geographical difference.

In summary, the *Sclerodermus* species reared in our laboratory belonged to three well-known species *S*. *guani*, *S*. *sichuanensis*, and *S*. *pupariae*, as well as five unidentified *Sclerodermus* populations, all of which were small and very similar morphologically. Because distinguishing these *Sclerodermus* species is difficult for many applied entomologists, we researched the differences between the five unidentified *Sclerodermus* species and the three well-known species by comparing different gene sequences to verify whether they were in accordance with the existing species-level identification or they were a new species. Firstly, on the basis of traditional morphological identification, which provided reliable evidence and very similar gene sequences *Sclerodermus* sp. (No. 4) was identified as *S*. *pupariae*. Secondly, although *S*. *guani* and *S*. *sichuanensis* have been published as two different species, our results showed 100% sequence indetity providing reliable evidence for revising the two species as one in the future. Thirdly, this is the first record of *Sclerodermus* sp. (No. 5) parasitizing *M*. *alternatus* larvae and pupae in *Pinus massoniana* Lamb. Further 95% of the female adults reared from one *M*. *alternatus* larvae were alate females, and the sequence data confirmed that this is a new species. *Sclerodermus* sp. (No. 2) was also first recorded in *Pinus massoniana* Lamb but parasitizing the larvae of *Chalcophora japonica*. Further, nearly all reared females were apterous. Thus, although *Sclerodermus* sp. (No. 2) and (No. 5) were found in *Pinus massoniana Lamb*, their hosts and morphological features differed; we could not clarify whether they are morphospecies. *Sclerodermus* sp. (No. 3) was first recorded parasitizing the larvae of *Chrysobothris* sp., which attacks *Larix gmelinii* in the north of China. Lastly, the sequences of *Sclerodermus* sp. (No. 1) were the most similar to those of *S*. *guani*, which parasitizes the pupae and larvae of *Semanotus sinoauster* Gressitt and *Saperda populnea* L. However, *Sclerodermus* sp. (No. 1) was first recorded parasitizing the pupae and larvae of *Agrilus mali*, which mainly attacks *Malus pumila Mill* and *Crataegi cuneatae*. Therefore, this species might have a new host. To arrive at the above conclusions, we have considered the host species, the tree species that the host attacks and morphological features to differentiate the unidentified species from the well-known species. A similar method was used for the first record of *Meteorus oviedoi* (Hymenoptera: Braconidae), a hymenopteran parasitoid of *Venadicodia caneti* (Lepidoptera: Limacodidae) in Costa Rica [30]. A previous study showed that combining several genetic characters may be the most practical way for efficient species identification [31]. In addition, we used molecular identification to verify our results obtained by traditional morphological identification.

Thus, although the gene sequences prove that *Sclerodermus* sp. (No. 5) is very likely a new species, and *Sclerodermus* sp. (No. 1), *Sclerodermus* sp. (No. 2), and *Sclerodermus* sp. (No. 3) are similar to the three well-known species, in our parallel research, we have been conducting other molecular analyses, such as ITS-1 and ITS-2, Cyt*b*, and ATP6 to verify whether there are any differences in the results.
